# Awareness, Knowledge, and Self-Reported Clinical Experiences Related to Glucose-6-Phosphate Dehydrogenase Deficiency in Sardinia (Italy): A Descriptive Cross-Sectional Survey

**DOI:** 10.3390/nu18111648

**Published:** 2026-05-22

**Authors:** Gabriele Serreli, Maria Paola Melis, Claudia Guerriero, Monica Deiana

**Affiliations:** Department of Biomedical Sciences, University of Cagliari, Cittadella Universitaria, SS 554, 09042 Monserrato, Italy

**Keywords:** awareness, cross-sectional study, dietary triggers, favism, fava beans, G6PD deficiency, health literacy, questionnaire, Sardinia

## Abstract

**Background:** Glucose-6-phosphate dehydrogenase (G6PD) deficiency is the most common enzymatic disorder of red blood cells, with particularly high prevalence in Sardinia, where it is strongly associated with favism. Public awareness remains incomplete and misconceptions persist—particularly regarding symptom onset from fava bean pollen or odors. This cross-sectional survey assessed G6PD self-reported deficiency, population knowledge, and persistence of false beliefs in Sardinia. **Methods:** A 16-item structured questionnaire was disseminated online (May–June 2025) to adults across diverse age groups and educational backgrounds. **Results:** Among 536 respondents (74.25% female; 97.39% Sardinia residents), 43.47% of respondents self-reported as G6PD-deficient, a figure substantially above the expected population estimate of 8–15% and consistent with affected-network recruitment. Moreover, 49.07% self-reported as non-deficient, and 7.46% were unaware of their status. While 99.07% correctly identified fava bean ingestion as a trigger and 74.25% identified certain medications, 62.50% incorrectly attributed hazard to pollen inhalation and 25.93% to pea consumption. Only 3.92% reported a hemolytic crisis, whereas 25.93% reported feeling unwell after smelling beans or inhaling pollen. Family and friends (49.81%) and healthcare providers (42.16%) were the primary information sources; schools (25.75%) and online resources (14.55%) were underrepresented. Overall, 90.45% perceived public information as insufficient—uniformly across G6PD strata (χ^2^ = 0.09, *p* = 0.955). Exploratory analyses suggested lower perceived information adequacy among younger respondents (Cochran–Armitage Z = 2.92, *p* = 0.002) and, less robustly, among female respondents (χ^2^ = 3.90, *p* = 0.048; borderline significance, unadjusted). **Conclusions:** Although recognition of fava bean ingestion as the principal dietary trigger is nearly universal, substantial gaps persist regarding non-ingestive exposures, less-recognized dietary triggers, and pharmacological risks. Perceived information insufficiency was independent of G6PD status but associated with younger age and female sex. Integrating targeted nutritional education into school curricula, primary care, and digital platforms is warranted for these priority groups and for G6PD-endemic populations worldwide.

## 1. Introduction

Glucose-6-phosphate dehydrogenase (G6PD) deficiency is the most common enzymatic disorder of red blood cells (RBC), affecting over 400 million people worldwide [1]. G6PD is a critical enzyme in the pentose phosphate pathway, which protects RBC from oxidative damage. Deficiency in this enzyme can lead to hemolytic anemia, particularly in response to certain drugs, infections, or foods such as fava beans (beans from *Vicia faba* species). For this reason, the clinical manifestation of this enzyme deficiency is also commonly called “favism” [2].

The distribution of G6PD deficiency is highly heterogeneous and closely mirrors historical malaria endemicity. High prevalence is observed in parts of Africa, the Middle East, South and Southeast Asia, and the Mediterranean region, where the condition is thought to have offered some protection against *Plasmodium falciparum* malaria throughout the centuries [3]. For example, in some African populations, prevalence can exceed 20%, while in Mediterranean countries like Greece and Italy, rates range from 3% to 15%. In Sardinia (Italy), G6PD deficiency affects approximately 8% to 15% of the male population, one of the highest rates in Europe [4].

The most common genetic variant in Sardinia is the G6PD Mediterranean variant (563 C > T), associated with markedly reduced enzyme activity and a higher risk of acute haemolytic episodes, especially after exposure to triggers such as fava beans or certain medications. Due to its X-linked inheritance, males are predominantly affected, although heterozygous females may also exhibit variable enzyme activity levels and clinical symptoms depending on X-chromosome inactivation patterns [5].

The primary cause of favism is the oxidative stress induced in RBC by fava beans, which contain high levels of vicine and convicine, compounds that are metabolised into reactive oxidants such as divicine and isouramil [6,7]. In G6PD-deficient individuals, the inability to regenerate reduced glutathione leads to RBC membrane damage and premature haemolysis.

Other ascertained contributing causes include concurrent infections, precarious health conditions and oxidative drugs (e.g., antimalarials), which may promote or exacerbate oxidative stress in RBC. The acute crisis in the G6PD-deficient usually presents within 24–48 h of exposure to triggering factors and includes symptoms like sudden onset of fatigue and weakness, jaundice, dark-colored urine (due to haemoglobinuria), pallor, abdominal or back pain, rapid heart rate and shortness of breath [8].

Although many individuals with G6PD deficiency remain asymptomatic throughout life, exposure to oxidative triggers can result in acute hemolytic episodes with clinically significant consequences. Hemolytic crises may require medical evaluation and, in severe cases, hospitalization and blood transfusion, particularly among infants and young children. Neonatal hyperbilirubinemia represents one of the most relevant complications and, if not promptly recognized and managed, may lead to kernicterus and long-term neurological sequelae [5]. Beyond the physical manifestations, G6PD deficiency may also impose a psychosocial burden on affected individuals and families. The need for lifelong vigilance regarding dietary exposures, infections, and medication safety can generate anxiety, especially among parents of affected children. Uncertainty surrounding potential triggers and the persistence of culturally transmitted beliefs may further contribute to restrictive behaviors and heightened risk perception, even in the absence of strong scientific evidence. These physical and psychological dimensions highlight the importance of accurate health information and effective educational strategies to support safe disease management while minimizing unnecessary fear and lifestyle limitations.

Despite decades of studies and research, G6PD deficiency is a genetic disease that remains little known by the population and for which there are still aspects to be explored from a clinical and informational standpoint. A notable example concerns the putative association between inhalation of fava bean pollen, or olfactory exposure to raw or cooked fava beans, and the onset of favism-related symptoms. Numerous anecdotal accounts have been reported, describing episodes of malaise in individuals passing through blooming fava bean fields or in proximity to freshly displayed fava beans. At present, however, no controlled study has established a mechanistic relationship between such non-ingestive exposures and the pathophysiology of G6PD deficiency [6]. Furthermore, lay understanding of the distinction between hemizygous males and heterozygous or, more rarely, homozygous females remains limited.

In Sardinia, information regarding G6PD deficiency is disseminated through multiple channels, although in a largely unstructured manner. Newborn screening for G6PD deficiency is widely implemented in regional healthcare settings, enabling early identification of affected individuals and facilitating initial counseling for families. However, beyond neonatal screening, Systematic public education programs specifically targeting G6PD deficiency are still underdeveloped. Information is most commonly conveyed through informal family transmission, reflecting the high prevalence of the condition and its longstanding recognition within the local cultural context. Healthcare professionals, particularly family physicians and pediatricians, represent another important source of individualized information, typically provided following diagnosis or during clinical encounters rather than through structured preventive initiatives.

In contrast, formal educational settings such as schools and universities rarely include comprehensive teaching on hereditary enzymatic disorders, including G6PD deficiency, despite their epidemiological relevance in Mediterranean populations. Public health campaigns and institutional awareness initiatives have been implemented only sporadically, often in response to local concerns or specific clinical events, resulting in heterogeneous knowledge across the population. Consequently, awareness of well-established triggers, such as fava bean ingestion, is generally high, whereas understanding of genetic transmission, pharmacological risks, and less substantiated triggers appears more variable. Characterizing the regional prevalence and molecular epidemiology of G6PD deficiency is essential for informing public health policies, particularly regarding newborn screening, drug safety, and dietary guidance. This is especially relevant in high-prevalence areas such as Sardinia, where the condition coexists with a cultural tradition of fava bean consumption. Against this background, we designed the present cross-sectional survey, enrolling more than 500 adult Sardinian residents, with the objectives of estimating the self-reported prevalence of G6PD deficiency, assessing the level of population knowledge, identifying persistent misconceptions, and informing the development of targeted health education strategies.

## 2. Materials and Methods

### 2.1. Sampling Technique

This cross-sectional survey was conducted by administering an online questionnaire distributed from 28 May 2025 to 10 June 2025 via multiple social media platforms (X, WhatsApp, Instagram, Facebook), targeting the general adult population of Sardinia. The recruitment strategy aimed to maximize demographic heterogeneity across age groups, educational levels, and G6PD status, without restriction to affected individuals (Figure S1 Participant flow diagram; Branch 1: Primary outcome analysis; Branch 2: Female enzyme-activity subgroup).

### 2.2. Study Design

The questionnaire was developed by the authors specifically for this study and comprised 16 items. These were developed based on a review of the literature and formulated in clear, accessible language to facilitate comprehension across different educational levels. The first section collected general demographic data; the second comprised three items on the self-reported epidemiology of G6PD deficiency in the Sardinian population; the third assessed respondents’ knowledge of G6PD deficiency, including triggering factors, associated symptoms, and recommended avoidance behaviors. Some items were mandatory and single-choice; others were optional (e.g., for women, whether they have G6PD deficiency and their enzyme activity grade) or allowed multiple responses. Prior to dissemination, the questionnaire was reviewed for clarity and face validity by all co-authors and by two independent lay readers with no medical background, in order to verify comprehensibility across different educational levels. No formal psychometric validation was performed, consistent with the exploratory nature of the study. Questionnaire items were classified a priori into three evidence categories: (A) established triggers or behaviours supported by robust scientific evidence (e.g., fava bean ingestion, specific medications including antimalarials and sulphonamides, infections); (B) items with preliminary or anecdotal evidence but lacking controlled mechanistic studies (e.g., inhalation of fava bean pollen, olfactory exposure to raw or cooked fava beans); and (C) items assessing common lay beliefs without established scientific support (e.g., pea consumption, tattoos) (Table S1). This classification informed the interpretation of responses and is indicated throughout the Results. All procedures were conducted in accordance with the ethical standards of the institutional and national research committee and with the 1964 Helsinki Declaration and its later amendments. Participation was entirely voluntary; respondents were informed that their answers would be fully anonymized and confidential. No personal identifiers were collected or stored. Data were recorded and analysed in aggregate form in compliance with the General Data Protection Regulation (EU Regulation 2016/679). The full questionnaire is provided as Supplementary File S1. This study is reported in accordance with the STROBE guidelines for cross-sectional studies (Supplementary Material S2).

### 2.3. Sample Size

**Figure 1:**
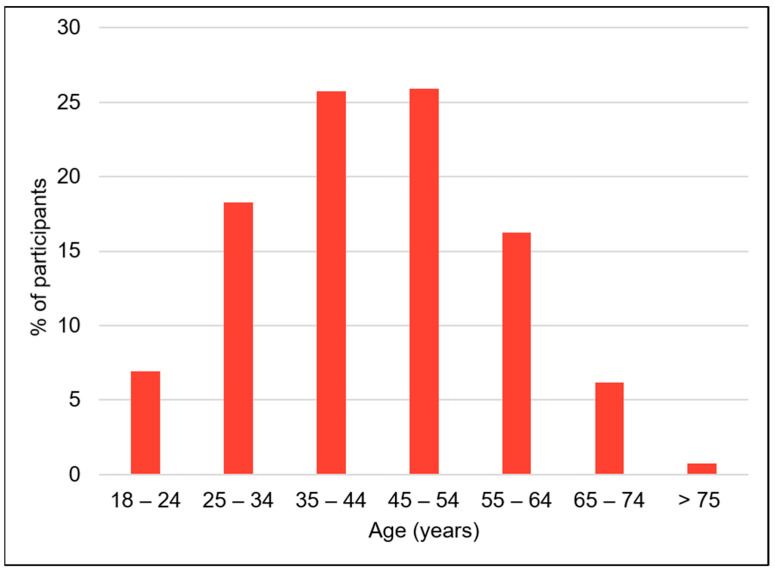
Breakdown of survey participants by age groups.

Sample size was estimated using the standard formula for single proportions (z = 1.96 for 95% confidence, assumed proportion *p* = 0.50, margin of error 5%), yielding a minimum of 385 participants for the reference Sardinian adult population of 1,558,119 inhabitants. Total participants at the end of the survey were 536, of which 398 female and 138 males. All participants are adults aged 18 to over 75 years old (Figure 1). We acknowledge that this formula assumes random sampling; given the convenience nature of recruitment via social media, the resulting figure should be interpreted as a minimum target for descriptive precision rather than a basis for population-level inference. All results are therefore reported as descriptive characteristics of the respondent sample and no claim of population representativeness is made.

### 2.4. Statistical Analysis

All analyses were performed on the full dataset of 536 respondents, stratified into three groups according to self-reported G6PD status: G6PD-deficient individuals (G6PD+, *n* = 233), non-deficient individuals (G6PD−, *n* = 263), and unknown status (*n* = 40). Descriptive statistics (absolute frequencies and percentages) were computed for all variables. Given the exploratory nature of the study, a single primary analysis was designated a priori: the association between self-reported G6PD status (three categories) and perceived information adequacy (χ^2^ test, df = 2, *n* = 534; two respondents were excluded due to missing data on this variable). All remaining analyses—including age- and sex-stratified comparisons, odds ratios, and subgroup percentages—are secondary and exploratory. No correction for multiple comparisons was applied; *p*-values should be interpreted accordingly.

To assess whether demographic characteristics and survey responses differed across the three strata, chi-square (χ^2^) tests of independence were applied to all categorical variables. Odds ratios (OR) with 95% confidence intervals (CI) were calculated from 2 × 2 contingency tables using unconditional maximum likelihood estimation with Wald 95% CI, comparing the odds of perceiving adequate public information on G6PD deficiency between strata, with the G6PD− stratum as reference. Age-stratified and gender-stratified analyses of awareness were conducted to explore potential effect modification. The association between age group and perception of adequate dissemination was tested using the chi-square test across all seven age groups (18–24, 25–34, 35–44, 45–54, 55–64, 65–74, ≥75 years). The survey instrument included items assessing respondents’ knowledge of G6PD-related triggers, specifically the correct identification of dietary and pharmacological agents to avoid. These knowledge items are reported separately as descriptive findings. The primary outcome for the analytical component of this study was operationally defined as perceived information adequacy: the proportion of respondents rating public information on G6PD deficiency as sufficient (“Yes” or “More yes than no”). It should be noted that this variable measures respondents’ subjective perception of information availability, not their objective knowledge of G6PD deficiency; findings regarding age and sex differences in this variable therefore reflect differential perceptions of information adequacy, not necessarily differential knowledge levels. A two-tailed *p*-value of <0.05 was considered statistically significant. All statistical analyses were performed using Python (version 3.12) with the pandas and SciPy libraries. To test the directional hypothesis that perceived information adequacy decreases with younger age, a Cochran–Armitage trend test was applied with age group treated as an ordinal variable (1 = 18–24 through 7 = ≥75 years).

## 3. Results

### 3.1. Demographic and Epidemiologic Results

Of the 536 study participants, 97.39% are residents of Sardinia, while the remaining 2.61% (14 participants) live elsewhere but are originally from Sardinia (Table 1).

**Figure 2:**
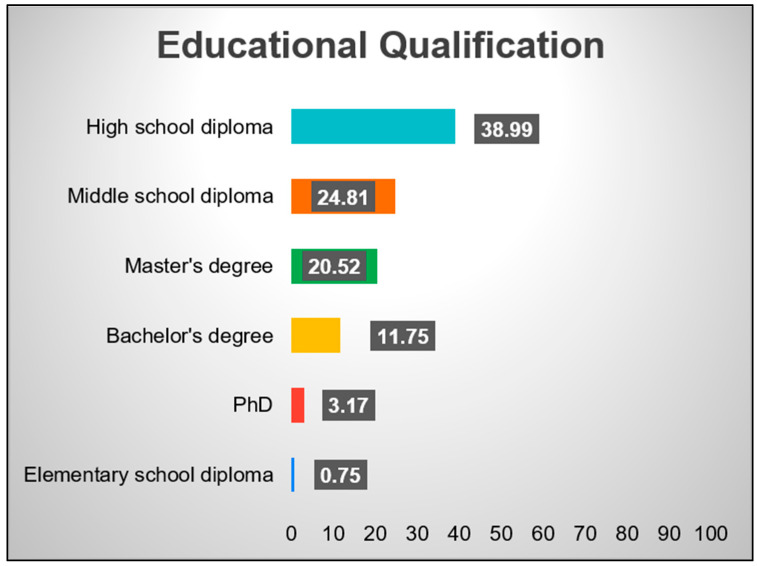
Percentages of participants’ educational qualifications, from elementary school to PhD (*n* = 536).

The distribution by age group shows that the participants involved were mainly between 35 and 44 years old (138 participants, 25.70%) and between 45 and 54 years old (139 participants, 25.93%). The population trend is approximately Gaussian (Figure 1). Of the respondents, 38.99% (*n* = 209) stated that they had a high school diploma as their highest educational qualification, while just over 30% stated that they had a bachelor’s degree (11.75%, *n* = 63) or a master’s degree (20.52%, *n* = 110). Next, 24.81% (*n* = 133) stated that they had a middle school diploma, while only 0.75% (*n* = 4) had only an elementary school diploma. Finally, there was also a small share of respondents who had a PhD as their highest qualification (3.17%, *n* = 17) (Figure 2). The educational profile of participants showed a relatively high proportion of individuals with secondary school and university degrees. This distribution may differ from that of the general Sardinian population, likely reflecting the online recruitment strategy. Regarding gender, 398 participants were female (74.25%) and 138 were male (25.75%). No statistically significant difference in gender distribution was observed across the three G6PD status strata (χ^2^ = 1.16, *p* = 0.56), nor was age distribution significantly different across strata (χ^2^ = 12.95, *p* = 0.37), indicating that the three groups were demographically comparable (Table 2). The distribution of G6PD status was as follows: 233 participants (43.47%) self-reported as G6PD-deficient (G6PD+), 263 (49.07%) as non-deficient (G6PD−), and 40 (7.46%) were unaware of their status (Table 3). Within each stratum, the proportions of males and females were similar: G6PD+ (75.54% female, 24.46% male), G6PD− (74.14% female, 25.86% male), and Unknown (67.50% female, 32.50% male) (Table 2). It should be noted that this classification is based entirely on respondent self-report and has not been confirmed by enzymatic assay or genetic testing; it should therefore be interpreted as respondent-perceived enzymatic status only, and no genotypic inference should be drawn from these figures.

When asked whether any relatives were G6PD-deficient, 63.06% (*n* = 338) affirmed having at least one affected family member, while 28.73% (*n* = 154) reported no affected relatives, and 8.21% (*n* = 44) were unaware (Table 3). As an optional item, female respondents were asked about their degree of G6PD deficiency; 200 out of 398 women (50.25%) responded. Of these, 24.00% (*n* = 48) reported complete deficiency, 46.50% (*n* = 93) partial deficiency, and 29.50% (*n* = 59) were unable to characterize their enzymatic status (Table 3).

### 3.2. Participants’ Awareness

**Figure 3:**
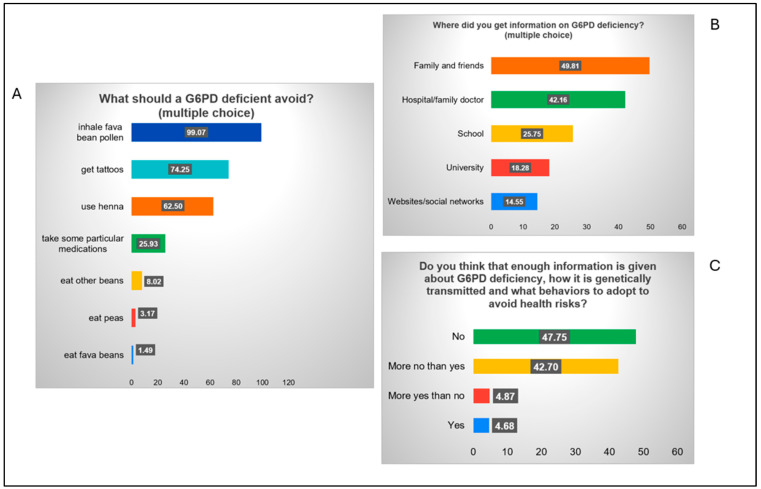
Percentages of answers to the questions “What should a G6PD deficient avoid? (multiple choice)” (**A**) (*n* = 536), “Where did you get information on G6PD deficiency? (multiple choice)” (**B**) (*n* = 536) and “Do you think that enough information is given about G6PD deficiency, how it is genetically transmitted and what behaviors to adopt to avoid health risks?” (**C**) (*n* = 534). Percentages in (**A**,**B**) sum to more than 100% as respondents could select multiple answers.

We asked some questions to understand the level of perception and knowledge of G6PD deficiency among the interviewed population. To the multiple-choice question “What should a G6PD deficient avoid?”, 99.07% (531 out of 536) of respondents answered “fava beans” (category A), while the second-most popular answer was “take some particular medications” with 74.25% (*n* = 398, Category A). In addition, 62.50% of respondents (*n* = 335) chose “inhale fava bean pollen” from the available answers (Category B). A further notable finding was the selection of peas as a potentially hazardous food by 25.93% of respondents (*n* = 139, Category C). By contrast, less frequently recognized avoidance behaviors included henna use (*n* = 43, 8.02%, Category B), consumption of other legumes (*n* = 17, 3.17%, Category C), and tattoos (*n* = 8, 1.49%, Category C) (Figure 3A).

We then asked where the respondents had obtained their information about G6PD deficiency, using a multiple-choice question. Briefly, 49.81% (*n* = 267) of the respondents said they had heard about favism in their family, while 42.16% (*n* = 226) had obtained information from their family doctor or a healthcare facility. Further down, only 25.75% said they had learned about it at school and 18.28% (*n* = 98) at university. Finally, only 14.55% (*n* = 78) said they had read something about it on websites and social networks (Figure 3B). Furthermore, with a specific question, we asked respondents “Do you think enough information is available about G6PD deficiency, how it is genetically transmitted, and what behaviors should be adopted to avoid health risks?”. Of the 534 respondents who answered this item, 255 (47.75%) answered ‘No’ and 228 (42.70%) answered ‘More no than yes’, indicating that 90.45% perceived public information as insufficient. Only 26 (4.87%) answered ‘More yes than no’ and 25 (4.68%) answered ‘Yes’, yielding a combined perceived-adequacy rate of 9.55% (*n* =  51/534) (Figure 3C). Notably, the distribution of responses to this question did not differ significantly across the three G6PD status strata (χ^2^ = 0.09, *p* = 0.955), indicating that perceived inadequacy of public information was similarly high regardless of whether respondents were G6PD-deficient, non-deficient, or unaware of their status. In the G6PD+ stratum, 9.91% perceived the available information as adequate (“Yes” or “More yes than no”), compared with 9.16% in the G6PD− stratum and 10.00% in the Unknown stratum (Table 4). The crude odds ratio for perceiving adequate information in the G6PD+ group relative to the G6PD− reference was 1.09 (95% CI: 0.60–1.99; *p* = 0.90), and 1.10 (95% CI: 0.36–3.36; *p* = 0.774) for the Unknown group. No association was detected within the precision of this sample; however, given *n* = 40 and a wide confidence interval, meaningful differences in perceived adequacy cannot be excluded (Table 5).

Age-stratified analysis revealed a statistically significant association between age group and perception of adequate public information (χ^2^ = 19.11, *p* = 0.004). Specifically, older respondents were more likely to perceive information as adequate: only 7.14–8.11% of participants aged 18–44 years rated public information as adequate, rising to 18.60% in the 55–64 age group and 21.21% among those aged 65–74 (Table 5). A Cochran–Armitage trend test confirmed a significant directional association between age and perceived information adequacy (Z = 2.92, *p* = 0.002, one-tailed), consistent with the hypothesis that younger respondents report lower perceived adequacy. Logistic regression with age group as an ordinal predictor yielded an odds ratio of 1.38 per age-band increase (95% CI: 1.11–1.72, *p* = 0.004), indicating that each successive age group was associated with a 38% increase in the odds of perceiving public information as adequate. This pattern was consistent across all three G6PD status strata. Gender-stratified analysis showed that male respondents were somewhat more likely to perceive information as adequate (13.77%) compared with female respondents (8.08%), with a slight statistically significant difference (χ^2^ = 3.90, *p* = 0.048).

### 3.3. Self-Reported Symptom Beliefs and Attributions

**Figure 4:**
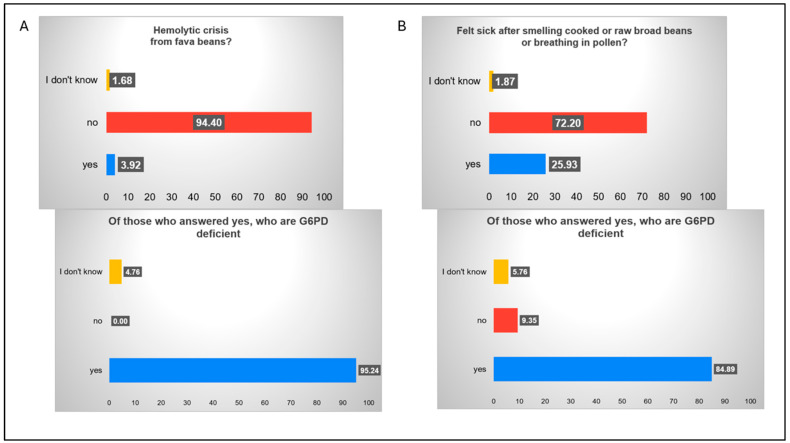
Percentage of answers to the questions “Have you ever had a fava bean hemolytic crisis?” (**A**) and “Have you ever felt sick after smelling cooked or raw fava beans or breathing in pollen?” (**B**). From these data, the selection of those who declared themselves to be G6PD deficient was then shown (*n* = 536).

**Figure 5:**
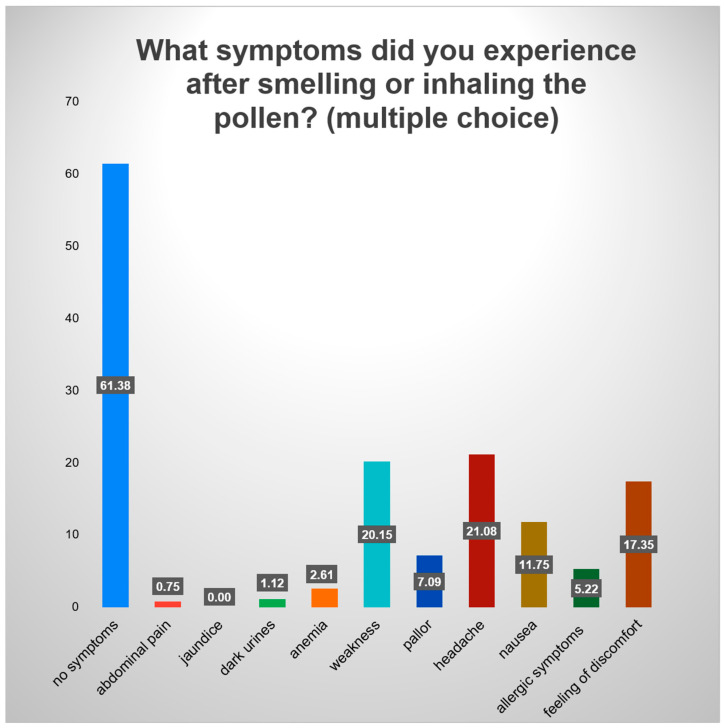
Percentage of answers to the question “What symptoms did you experience after smelling or inhaling the pollen? (multiple choice)” including both G6PD and non-G6PD-deficient individuals.

**Figure 6:**
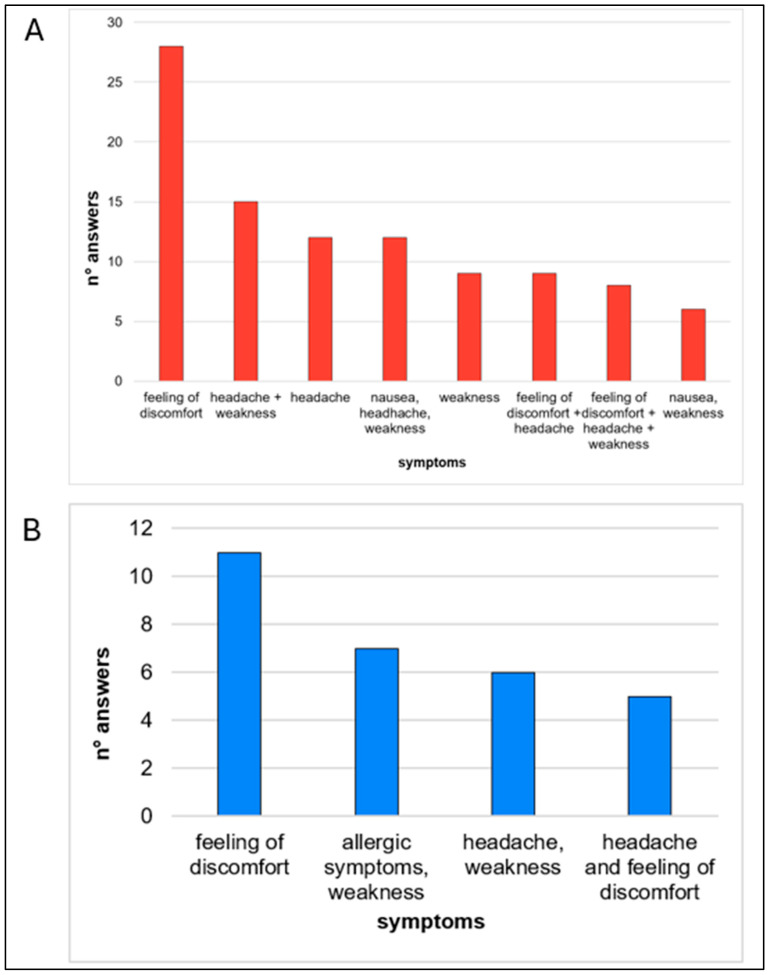
Symptoms mostly present in G6PD-deficient individuals (**A**) and in non-G6PD-deficient individuals (**B**) after pollen inhalation or smelling cooked fava beans. Combinations of symptoms found in at least 5 questionnaires were taken into consideration.

The following data reflect respondents’ retrospective self-attribution of symptoms to specific exposures. No information was collected on timing, dose, co-exposures, or biochemical confirmation; these data therefore cannot be used to establish causal associations or to classify events as clinically confirmed haematological outcomes. Only 3.92% of all respondents reported having experienced a hemolytic crisis following fava bean ingestion (Figure 4A, Category A); of these, 95.24% identified as G6PD-deficient individuals and 4.76% were unaware of their status. When asked whether they had ever felt unwell after smelling raw or cooked fava beans or inhaling fava bean pollen, approximately one in four respondents (25.93%, *n* = 139/536, Category B) answered affirmatively; of these, 84.89% self-reported as G6PD-deficient individuals. Whether these symptoms reflect genuine haematological events or non-specific discomfort attributable to other causes cannot be determined from self-report data alone (Figure 4B). We then evaluated other possible symptoms potentially linked to the discomfort experienced following inhalation of fava bean pollen or the odor of raw or cooked fava beans using a multiple-choice question. Approximately 61% of all the respondents, including both G6PD-deficient and non-G6PD-deficient individuals, stated that they had not experienced any problems following exposure to fava beans or their pollen. Twenty-one percent of respondents reported having headache; 20.15% reported weakness, 17.35% reported feeling uncomfortable, and 11.75% experienced nausea. To a lesser extent, respondents reported pallor (7.09%), allergic symptoms (5.22%), anemia (2.61%), dark urine (1.12%), jaundice (0.75%), and abdominal pain (0.75%) (Figure 5). Among G6PD-deficient individuals, twenty-eight respondents reported feeling unwell, then simultaneously reported headache and weakness, twelve reported headaches, and the same number reported a combination of headache, nausea, and weakness. Among non-G6PD-deficient individuals, eleven respondents reported feeling unwell, seven reported weakness and allergic symptoms simultaneously, six reported headache and weakness simultaneously, and five reported headache and malaise (Figure 6).

**Figure 7:**
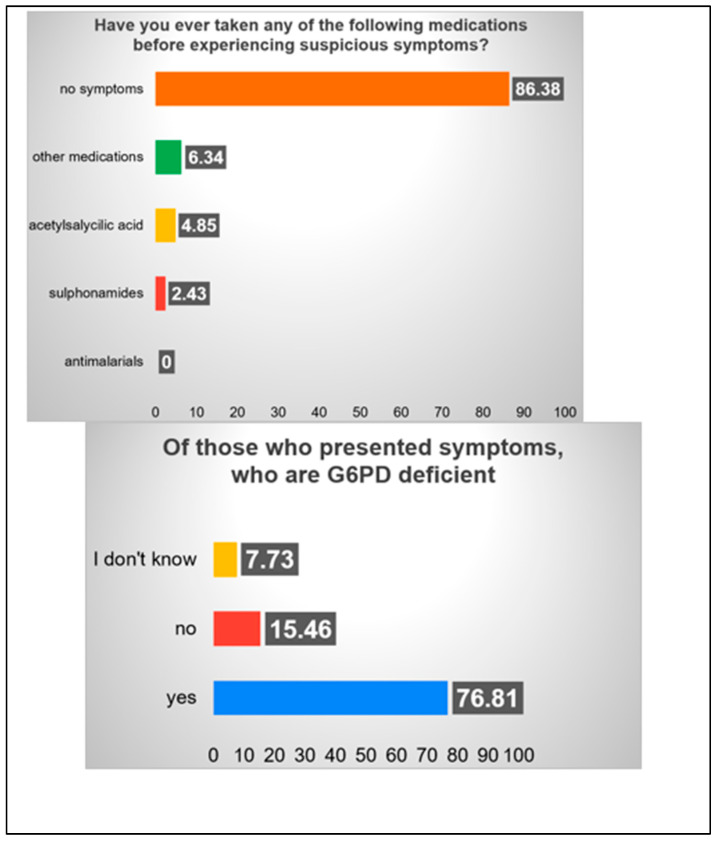
Percentage of answers to the question “Have you ever taken any of the following medications before experiencing suspicious symptoms like those listed above?” and selection of results related to G6PD-deficiency patients (*n* = 536).

We contextually asked respondents the multiple-choice question “Have you ever taken any of the following medications before experiencing suspicious symptoms like those listed above?”. Most of respondents (86.38%) stated they had never experienced symptoms attributable to favism after taking certain medications, while the remaining 13.62% experienced problems after taking acetylsalicylic acid (4.85%, Category A), sulphonamides (2.43%, Category A), or other unspecified medications (6.34%). Among the respondents that showed symptoms after taking medications, 64.38% were G6PD-deficient individuals, 27.40% were not, and 8.22% stated they did not know (Figure 7).

## 4. Discussion

The present investigation provides an in-depth examination of the demographic, epidemiologic, awareness, and clinical dimensions of G6PD deficiency within a Sardinian population cohort. With 536 respondents, the findings highlight both the prevalence of the condition and the widespread perception of its clinical implications, while also underscoring significant gaps in knowledge and information dissemination.

### 4.1. Demographic Profile

The demographic characteristics of the study population are partly influenced by the unique epidemiological setting of Sardinia, where G6PD deficiency is highly prevalent. However, due to convenience sampling, these data are not representative of the general population. Nearly all respondents were Sardinian residents, and even the few non-residents claimed to come from Sardinia, reflecting the high prevalence of G6PD deficiency and its recognition within the local cultural context of Sardinia. The age distribution of participants, primarily concentrated between 35 and 54 years, reflects an adult population that is both aware of and affected by health issues linked to hereditary conditions. Gender representation was skewed toward females, which may partly reflect greater willingness to participate in health-related surveys, a phenomenon observed in other epidemiological studies [9]. Within each G6PD status stratum, age and gender distributions were similar and did not differ significantly across strata, suggesting that the stratified comparisons are not confounded by major demographic imbalances. The proportion of individuals declaring themselves G6PD deficient is higher than previously reported high prevalence rates in Sardinia [10]. Interestingly, while nearly half reported no personal deficiency, more than 60% acknowledged having at least one relative with the mutation. This highlights the familial clustering typical of X-linked hereditary conditions and underscores the awareness of G6PD deficiency as a shared familial trait. Among women, partial self-reported deficiency was the most frequent reported degree of enzyme deficiency. However, the fact that nearly one-third of female respondents were unable to characterize their enzymatic status is notable, as it reflects a diagnostic gap that could have important implications for reproductive counseling and risk assessment.

### 4.2. Knowledge and Misconceptions

Despite widespread recognition of the link between fava beans and hemolytic crises, the findings reveal substantial misconceptions and incomplete knowledge regarding triggers and preventive measures. For instance, over 60% of respondents incorrectly identified fava bean pollen as a hazardous exposure. While some anecdotal reports and individual experiences suggest possible reactions to pollen [6], robust scientific evidence remains limited. The high prevalence of this belief underscores the need for clearer communication from healthcare providers to distinguish between well-established and less substantiated risk factors. Another significant proportion of respondents—approximately one in four—indicated that consuming peas or other legumes may be associated with symptoms of favism. This misconception may be associated with historically documented practices of co-cultivating peas and fava beans in the same fields, which could result in cross-contamination during harvesting and packaging and thus accidental fava bean ingestion [11]. It is well established that pea consumption is safe for G6PD-deficient individuals, and no cases of favism attributable to pea ingestion have been documented in the literature [6,12]. Of particular interest, less than half of respondents cited healthcare professionals or facilities as their primary source of information, while nearly half relied on family narratives. This reliance on informal sources may perpetuate misconceptions and highlights a gap in structured health education. Moreover, only one in four respondents reported receiving information at school and fewer than one in five at university. This suggests that formal education on hereditary enzymatic disorders is still insufficient, despite their relatively high prevalence in certain Mediterranean regions. These findings align with previous research demonstrating limited awareness of G6PD deficiency among lay populations, even in high-prevalence areas [13]. Importantly, nine of ten respondents expressed dissatisfaction with the availability of adequate information, reinforcing the urgency of developing targeted educational interventions. From an analytical standpoint, several noteworthy patterns emerged when stratifying the data by G6PD status, age, and gender. Crucially, no statistically significant difference in the perception of inadequate public information was detected across the three G6PD status strata, with odds ratios close to unity—a pattern consistent with the interpretation that this concern extends beyond those personally affected. However, given the small size of the Unknown stratum (*n* = 40) and the correspondingly wide confidence interval, a definitive conclusion of equivalence across all strata cannot be drawn. This pattern is also consistent with the rationale for population-level awareness campaigns, though replication in larger samples—particularly with adequate representation of individuals of unknown G6PD status—would be needed to support this conclusion more firmly. The age-stratified analysis revealed a significant gradient: younger respondents (aged 18–44) consistently rated available information as inadequate at rates above 90%, while older age groups—particularly those aged 55–74—showed relatively higher, though still modest, satisfaction with available information. This age-related pattern may reflect generational differences in health expectations, greater exposure to digital health content among younger cohorts, or a more critical appraisal of available resources, rather than necessarily indicating that younger respondents receive objectively less information. It also suggests that young adults represent the group reporting the greatest perceived inadequacy of public information, warranting prioritized outreach. Gender-stratified analysis revealed that male respondents were, in a slightly significant way, more likely to perceive public information as adequate compared with female respondents. Whether this reflects lower objective knowledge, higher expectations, or greater critical awareness of information gaps cannot be determined from the present data, which measure perceived adequacy rather than verified knowledge. If confirmed in larger, more balanced samples, this pattern might reflect differential access to diagnosis or counseling, including the possibility that males are more often identified through neonatal screening programmes. Given the borderline *p*-value (*p* = 0.048), the absence of multiplicity correction across subgroup analyses, and the asymmetric sex composition of the sample, this finding should be considered hypothesis-generating and requires replication before causal interpretation is warranted. Moreover, it should be noted that several subgroup estimates rest on small cell sizes—notably the ≥75 years group (*n* = 4) and the Unknown G6PD stratum (*n* = 40)—which limits their statistical precision. These findings should accordingly be treated as hypothesis-generating rather than confirmatory.

### 4.3. Self-Reported Clinical Experiences

Clinical experiences reported by respondents illustrate both expected and notable patterns. Only a small percentage reported hemolytic crises following fava bean ingestion, despite nearly half identifying as G6PD-deficient individuals. This low frequency may reflect effective avoidance behaviors, consistent with widespread awareness of the principal dietary risk. In contrast, over one in four respondents reported adverse symptoms following exposure to fava bean odors or pollen. Although most of these individuals identified as G6PD-deficient, the pathophysiological basis of such reactions remains uncertain. As stated previously, a small number of preliminary and methodologically limited case reports have described hemolytic episodes following inhalation exposure [14], yet larger and more reliable clinical studies are lacking. Thus, while such self-reported experiences should not be dismissed, further controlled investigations are warranted to clarify whether these symptoms represent true hemolytic events or non-specific discomfort. Symptom reports following inhalation exposure included headaches, weakness, malaise, and nausea, with very small proportions reporting pallor, anemia, or dark urine. Interestingly, symptom clusters differed between G6PD-deficient and non-deficient respondents, suggesting that perception of susceptibility may influence symptom reporting. For instance, while twenty-eight G6PD-deficient respondents reported concurrent headache and weakness, non-deficient respondents more commonly associated weakness with allergic manifestations. Such differences point up the interplay between genetic predisposition, environmental triggers, and subjective symptom perception. Respondents with a family history of G6PD deficiency or prior concern about the condition may be more likely to attribute non-specific symptoms to perceived exposure, a phenomenon consistent with symptom expectancy or nocebo effects. This possibility should be considered when interpreting the 25.93% reporting of feeling unwell after smelling fava beans or inhaling pollen, and the finding should not be taken as evidence of a verified physiological response to olfactory exposure.

Regarding pharmacological exposures, only 13.62% reported adverse effects after taking medications known to precipitate hemolysis in G6PD-deficient individuals. Aspirin and sulphonamides were most commonly implicated, consistent with established literature [15]. It should be noted that acetylsalicylic acid at standard therapeutic doses is generally considered safe in G6PD-deficient individuals [6]; symptoms reported here following aspirin use may therefore reflect misattribution to medication, concomitant exposure to other triggers, or supratherapeutic dosing. It is also possible that some respondents confused acetylsalicylic acid with other NSAIDs or with combination cold and flu preparations containing oxidative agents, which would further complicate interpretation of self-reported medication awareness. Finally, the fact that a minority of non-deficient respondents also reported symptoms may reflect either misattribution or unrecognized subclinical deficiency.

### 4.4. Public Health Implications

The findings of this study carry several important implications for public health policy, clinical and nutritional practice, and community education in regions with high G6PD-deficiency prevalence, including but not limited to Sardinia. Such patterns of limited awareness and persistent misconceptions have also been documented in other high-prevalence settings, including sub-Saharan Africa, the Middle East, and Southeast Asia, where G6PD deficiency disproportionately affects local populations [3,16]. First, the perceived gaps in available information—particularly regarding lesser-known triggers and genetic transmission—stress the importance of strengthening educational outreach. Specifically, a single dedicated session within lower-secondary school biology curricula—covering G6PD deficiency, its X-linked inheritance, and the principal dietary and pharmacological triggers—paired with a take-home information sheet for parents, could serve as a low-cost, high-reach intervention in G6PD-endemic regions such as Sardinia. This approach would simultaneously target adolescents at a key educational stage and their families, who represent the primary information network identified in this study. Second, the discrepancy between self-reported and biochemically confirmed prevalence underscores the need for confirmatory enzymatic or genetic testing. Systematic screening—whether through expanded neonatal programs or targeted community initiatives—would improve epidemiological mapping and enable earlier identification of at-risk individuals. Integration of G6PD testing into routine healthcare frameworks could support earlier diagnosis, facilitate personalized counseling, and inform safe pharmacological decision-making. Third, the comparatively low incidence of hemolytic crises relative to the widespread perception of risk is consistent with largely effective avoidance behaviors. However, such behaviors may also be associated with unnecessary anxiety and disproportionate lifestyle restrictions. The psychosocial burden of G6PD deficiency merits particular attention. The persistent belief that inhalation of fava bean pollen or other non-ingestive exposures may trigger hemolysis may generate unwarranted fear and overly restrictive behavioral responses. Healthcare providers are well-positioned to address these concerns through balanced, evidence-based communication—reinforcing established avoidance measures while offering appropriate reassurance regarding risk factors that lack robust scientific support. From a clinical standpoint, increased vigilance is justified among prescribers in high-prevalence regions. Routine verification of G6PD status prior to administering drugs with oxidative potential, supported by automated alerts in electronic prescribing systems and strengthened pharmacovigilance networks, could reduce preventable adverse drug reactions. The substantial proportion of “I don’t know” responses constitutes an important indicator of diagnostic uncertainty within the population. This pattern may reflect both knowledge gaps and limited access to confirmatory testing, particularly among women, in whom heterozygous status is associated with variable clinical expression and less frequent diagnostic evaluation. Such uncertainty may be associated with inconsistent risk perception and preventive behavior. From a public health perspective, these respondents represent a priority target for educational outreach, with emphasis on genetic transmission, diagnostic options, and individualized risk communication.

### 4.5. Limitations

Several limitations should be acknowledged. The study relied on self-reported data, which may be subject to recall bias and misclassification, particularly regarding enzymatic status and clinical symptoms. The elevated proportion of self-reported G6PD-deficient respondents (43.47%)—substantially higher than the estimated population prevalence of 8–15% in Sardinian males—together with the high proportion of participants reporting an affected relative (63.06%) and the marked overrepresentation of female respondents (74.25%), strongly suggests that recruitment occurred predominantly through affected-family and social networks rather than representing a population-based sample. This pattern of affected-network enrichment is a recognized feature of online health surveys and should be considered when interpreting the self-reported prevalence figures, which reflect the composition of the respondent sample and cannot be generalized to the Sardinian population. Accordingly, all findings are presented as descriptive of a self-selected, condition-enriched convenience sample. In this context, another aspect to consider is that the educational level of participants was relatively high compared with the general population, which is a common feature of online survey-based studies [17]. This may have led to an overestimation of perceived information adequacy, as individuals with higher education are generally more likely to engage with health information and participate in research. Consequently, dissatisfaction with available information observed in this study may be even more pronounced in less educated population groups. This potential selection bias should be considered when interpreting the generalizability of the findings. Moreover, the lack of biochemical confirmation of G6PD status limits the ability to directly correlate self-reported symptoms with true deficiency. Finally, another limitation is the lack of formal validation of the questionnaire, which may affect measurement precision and comparability with other studies. Review by co-authors and lay readers, while useful for establishing face validity, does not constitute formal psychometric validation of the instrument. Item-level reliability, construct validity, and test–retest stability were not assessed. Formal validation—including pilot testing with inter-item agreement measures—is a requirement for future waves of this questionnaire.

Post-stratification weighting using ISTAT Sardinia population denominators was here considered but not applied, as demographic weighting would correct for age and sex composition without addressing the network-driven overrepresentation of G6PD-positive individuals inherent in convenience sampling. Probability-based sampling combined with post-stratification weighting is recommended for future waves. Nevertheless, the instrument focused on straightforward factual questions, reducing the likelihood of substantial misinterpretation. Moreover, future research should pursue more rigorous methodological approaches to consolidate these findings. Longitudinal designs or controlled challenge studies incorporating exposure–symptom diaries could clarify the physiological plausibility of inhalational triggers. Qualitative investigations exploring the cultural and familial transmission of G6PD-related beliefs would illuminate how misinformation is perpetuated within local communities. Comparative cross-national studies examining awareness and misconceptions in other high-prevalence populations—such as those in West Africa, the Arabian Peninsula, or Southeast Asia—would contextualize the present findings within a broader international framework and inform the development of region-specific educational interventions. Finally, intervention studies evaluating structured health education programs would determine whether targeted communication strategies can effectively enhance knowledge, correct misconceptions, and promote evidence-based self-management behaviors.

## 5. Conclusions

In conclusion, this study provides evidence-based insights into the awareness and clinical correlates of G6PD deficiency in a high-prevalence Mediterranean region. While recognition of fava bean ingestion as the principal dietary trigger is nearly universal, substantial knowledge gaps persist regarding less-recognized dietary and non-dietary triggers, X-linked inheritance, and pharmacological risks. Critically, perceived information insufficiency was uniform across all G6PD strata and significantly more pronounced among younger respondents and women—identifying these as priority groups for proactive, population-level educational interventions. These findings are broadly applicable to other G6PD-endemic populations worldwide, where the intersection of traditional food practices and enzymatic vulnerability creates analogous public health challenges.

## Figures and Tables

**Table 1:** Demographic characteristics of study participants.

Variable		Frequency	Percentage
Gender	Male	138	25.75
	Female	398	74.25
	Total	536	100.00
Resident in Sardinia	Yes	521	97.39
	Not resident but originally from Sardinia	14	2.61
	Total	536	100.00

**Table 2:** Demographic Characteristics of Study Participants (*n* = 536).

Variable	Category	G6PD+ (*n* = 233)	G6PD− (*n* = 263)	Unknown (*n* = 40)	Total (*n* = 536)	*p*-Value
Gender	Female	176 (75.54%)	195 (74.14%)	27 (67.50%)	398 (74.25%)	χ^2^ = 1.16 *p* = 0.56
	Male	57 (24.46%)	68 (25.86%)	13 (32.50%)	138 (25.75%)	
Age group (years)	18–24	9 (3.86%)	25 (9.51%)	3 (7.50%)	37 (6.90%)	χ^2^ = 12.95 *p* = 0.37
	25–34	46 (19.74%)	43 (16.35%)	9 (22.50%)	98 (18.28%)	
	35–44	56 (24.03%)	71 (27.00%)	11 (27.50%)	138 (25.75%)	
	45–54	66 (28.33%)	65 (24.71%)	8 (20.00%)	139 (25.93%)	
	55–64	44 (18.88%)	37 (14.07%)	6 (15.00%)	87 (16.23%)	
	65–74	11 (4.72%)	19 (7.22%)	3 (7.50%)	33 (6.16%)	
	>75	1 (0.43%)	3 (1.14%)	0 (0.00%)	4 (0.75%)	

Note: Values expressed as *n* (%). *p*-values from chi-square tests. G6PD+ = G6PD deficient; G6PD− = non-deficient; Unknown = self-reported unknown status.

**Table 3:** Epidemiologic statistics.

Variable		Frequency	Percentage
	Yes	233	43.47
Are you G6PD deficient?	No	263	49.07
	I don’t know	40	7.46
	Total	536	100.00
	Complete deficiency (self-reported)	48	24.00
female grade of deficiency (optional)	Partial deficiency (self-reported)	93	46.50
	I don’t know	59	29.50
	Total	200	100.00
	Yes	338	63.06
Any relative with G6PD deficiency?	No	154	28.73
	I don’t know	44	8.21
	Total	536	100.00

Note: female grade of deficiency was optional, *n*  =  200/398 female respondents, 198 not answered. Labels reflect respondents’ self-reported degree of deficiency as indicated in the questionnaire item; genotype cannot be inferred from self-report. The terms “complete deficiency” or “partial deficiency” do not imply molecular or enzymatic confirmation.

**Table 4:** Perception of Adequate Information on G6PD Deficiency, by G6PD Status.

Response	G6PD+ (*n* = 232)	%	G6PD− (*n* = 262)	%	Unknown (*n* = 40)	%	Total (*n* = 534)
Yes	10	4.31%	13	5.00%	2	5.00%	25 (4.68%)
More yes than no	13	5.60%	11	4.20%	2	5.00%	26 (4.87%)
More no than yes	92	39.66%	118	45.00%	18	45.00%	228 (42.70%)
No	117	50.43%	120	45.80%	18	45.00%	255 (47.75%)
Total	232	100.00%	262	100.00%	40	100.00%	534
Chi-square test across strata: χ^2^ = 0.09, df = 2, *p* = 0.955|Perceived adequate information (Yes + More yes): G6PD+ 9.91%, G6PD− 9.16%, Unknown 10.00%							

Note: Question asked: ‘Do you think there is enough public information on G6PD deficiency, its genetic transmission, and how to avoid health risks?’ Two participants did not respond to this item; analyses are based on *n* = 534 (G6PD+: *n* = 232; G6PD−: *n* = 262; Unknown: *n* = 40).

**Table 5:** Analytical Results: Perceived Information Adequacy by Age Group, Gender and G6PD Status.

A. Perception of Adequate Information by Age Group and G6PD Status								
Age Group	G6PD+ (*n*)	% Perceived Adequate	G6PD− (*n*)	% Perceived Adequate	Unknown (*n*)	% Perceived Adequate	All Strata (*n*)	% Perceived Adequate (Combined)
18–24	9	11.11%	25	8.00%	3	0.00%	37	8.11%
25–34	46	6.52%	43	6.98%	9	11.11%	98	7.14%
35–44	55	7.27%	71	7.04%	11	9.09%	137	7.30%
45–54	66	4.55%	65	4.62%	8	12.50%	139	5.04%
55–64	44	22.73%	36	16.67%	6	0.00%	86	18.60%
65–74	11	18.18%	19	21.05%	3	33.33%	33	21.21%
>75	1	0.00%	3	33.33%	0	-	4	25.00%
Trend across age groups (all strata combined): χ^2^ = 19.11, *p* = 0.004 → Older age groups show significantly higher perception of adequate information. Cochran–Armitage trend test confirmed a significant directional association between age and perceived information adequacy (Z = 2.92, *p* = 0.002, one-tailed). Logistic regression with age group as an ordinal predictor yielded an odds ratio of 1.38 per age-band increase (95% CI: 1.11–1.72, *p* = 0.004).								
**B. Perception of Adequate Information by Gender and G6PD Status**								
**Gender**	G6PD+ (*n*)	% Perceived Adequate	G6PD− (*n*)	% Perceived Adequate	Unknown (*n*)	% Perceived Adequate	All strata (*n*)	% Perceived Adequate (combined)
**Female**	175	8.57%	194	7.73%	27	7.41%	396	8.08%
**Male**	57	14.04%	68	13.24%	13	15.38%	138	13.77%
**Gender difference (all strata combined): χ^2^ = 3.90, *p* = 0.048 → Males show higher perception of adequate information (13.77% vs. 8.08%)**								
**C. Odds Ratios for Perceived Adequate Information (Reference: G6PD− Stratum)**								
**Comparison**	**Crude OR**	**95% CI Lower**	**95% CI Upper**	**Chi-Square**	***p*** **-Value**	**Interpretation**		
**G6PD+ vs. G6PD−**	1.09	0.60	1.99	0.02	0.8956	No difference detected; estimate imprecise		
**Unknown vs. G6PD−**	1.10	0.36	3.36	0.00	1.0000	No difference detected; estimate imprecise (wide CI, *n* = 40)		

Note: OR = Odds Ratio; 95% CI computed from 2 × 2 contingency tables using unconditional maximum likelihood estimation with Wald 95% CI. Perceived information adequacy = ‘Yes’ or ‘More yes than no’ responses. Two participants did not respond to this item; analyses are based on *n* = 534 (G6PD+: *n* = 232; G6PD−: *n* = 262; Unknown: *n* = 40).

## Data Availability

The raw data supporting the conclusions of this article will be made available by the authors on request.

## Supplementary Materials

The following supporting information can be downloaded at: https://www.mdpi.com/article/10.3390/nu18111648/s1. Figure S1: Participant flow diagram; Branch S1: Primary outcome analysis; Branch S2: Female enzyme-activity subgroup; Table S1: Item-level Response Completeness; File S1: Full Questionnaire; Material S2: STROBE Checklist for Cross-Sectional Studies [18].

## Abbreviations

The following abbreviations are used in this manuscript:

G6PD	Glucose-6-phosphate dehydrogenase
RBC	Red blood cells
