# Supplementary material for: Awareness, Knowledge, and Self-Reported Clinical Experiences Related to Glucose-6-Phosphate Dehydrogenase Deficiency in Sardinia (Italy): A Descriptive Cross-Sectional Survey
Source: Nutrients. 2026 May 22;18(11):1648. doi: 10.3390/nu18111648 (PMC13258678; doi:10.3390/nu18111648)
Supplement: Supplementary file 1 [file nutrients-18-01648-s001.zip › nutrients-4311563-supplementary.pdf]

Supplementary Material

Awareness, knowledge, and self-reported clinical experiences related to glucose-6-phosphate dehydrogenase (G6PD) deficiency in Sardinia (Italy): a descriptive cross-sectional survey

Gabriele Serreli[1*], Maria Paola Melis[1], Claudia Guerriero[1] & Monica Deiana[1]

[1]Department of Biomedical Sciences, University of Cagliari, Cittadella Universitaria SS 554, Monserrato, 09042, Italy

*Corresponding author. E-mail address: gabriele.serreli@unica.it; tel. 0706754185

Supplementary Figure S1. Participant Flow Diagram

STROBE-style diagram showing the flow of participants from survey responses through to each analytical population. Numbers are derived from the raw dataset (N = 536 total respondents).

	**Individuals who responded to the online survey**<br>(N = 536)	
	▼	
	**Excluded: duplicate/test responses**<br>(n = 0)	
	▼	
	**Included in descriptive analysis**<br>(N = 536)	
	▼	

Analytical sub-populations:

Branch S1 — Primary outcome analysis (perceived information adequacy):

	**Primary outcome analysis**<br>Perceived information adequacy<br>(n = 534; 2 missing on Q16)		**Excluded from Q16 analysis**<br>Missing response on item 16<br>(n = 2)	
	▼			
	**χ^2 primary analysis**<br>G6PD status × perceived adequacy<br>G6PD-deficient: n=233 ǀ Non-deficient: n=263 ǀ Unknown: n=40<br>(n = 534; df = 2)			

Branch S2 — Female enzyme-activity subgroup (optional item):

	**Female respondents**<br>(n = 398)			
	▼			

	**Completed optional Q8**<br>(enzyme activity grade)<br>n = 200 / 398 answered<br>n = 198 did not answer (not applicable or left blank)			

Note: G6PD-deficient individuals: n = 233 (self-reported Yes); non-deficient: n = 263 (No); unknown status: n = 40 (I don't know). All G6PD status classifications are based entirely on self-report and have not been confirmed by enzymatic assay or genetic testing.

Figure abbreviations: Q8 = optional enzyme-activity question (female G6PD-deficient respondents only); Q16 = perceived information adequacy item (4-point scale: Yes / More yes than no / More no than yes / No).

Supplementary Table S1. Item-level Response Completeness

Completeness of responses for each of the 16 questionnaire items (N = 536 respondents). Items are classified as mandatory (all respondents) or optional (female G6PD-deficient respondents only, Q8). Valid responses include all non-blank entries regardless of specific answer chosen.

Item	Description (English)	Status	Valid (n)	Missing (n)	Completion (%)
**Q1**	Sex	Mandatory	536	0	100.0%
**Q2**	Education level	Mandatory	536	0	100.0%
**Q3**	Age group	Mandatory	536	0	100.0%
**Q4**	Province of residence	Mandatory	536	0	100.0%
**Q5**	Sardinia residence >10 years	Mandatory	536	0	100.0%
**Q6**	Ethnic/geographic origins	Mandatory	536	0	100.0%
**Q7**	Self-reported G6PD status (Yes/No/Unknown)	Mandatory	536	0	100.0%
**Q8**	Female enzyme-activity grade (intermediate/total/unknown) [optional]	Optional	200	198	50,3%
**Q9**	Affected family member (favism/G6PD deficiency)	Mandatory	536	0	100.0%
**Q10**	Behaviours to avoid (multiple-choice knowledge item)	Mandatory	536	0	100.0%
**Q11**	Ever had a haemolytic crisis after fava bean ingestion	Mandatory	536	0	100.0%
**Q12**	Ever felt unwell after fava bean odour/pollen inhalation	Mandatory	536	0	100.0%
**Q13**	Symptoms after odour/pollen exposure (multiple-choice)	Mandatory	536	0	100.0%
**Q14**	Suspected drug reactions (prior medication use)	Mandatory	536	0	100.0%
**Q15**	Source(s) of information on favism (multiple-choice)	Mandatory	526	10	98.1%
**Q16**	Perceived adequacy of public information (4-point scale)	Mandatory	534	2	99.6%

Highlighted row (Q8, yellow): optional item administered only to female respondents who self-identified as G6PD-deficient (n = 200/398 female respondents answered; n = 198 left blank or item not applicable).

Supplementary File S1. Full Questionnaire (Italian Original with English Translation)

The survey was developed by the authors and administered online via Google Forms. Prior to dissemination, the questionnaire was reviewed for clarity and face validity by all co-authors and by two independent lay readers with no medical background.

Items were classified a priori into three evidence categories:

[A] Established triggers or behaviours supported by robust scientific evidence (e.g., fava bean ingestion, specific medications, infections).

[B] Items with preliminary or anecdotal evidence but lacking controlled mechanistic studies (e.g., pollen inhalation, olfactory exposure).

[C] Items assessing common lay beliefs without established scientific support (e.g., pea or legume consumption, tattoos).

Category codes are indicated in the 'Cat.' column and within response options for Q10.

Item	Cat.	Italian (original)	English (translation)
**Q1**	**A**	**Genere**<br>*Response options: Femmina / Maschio*	**Sex**<br>*Response options: Female / Male*
**Q2**	**A**	**Titolo di studio**<br>*Response options: Licenza elementare / Licenza media / Diploma scuola superiore / Laurea triennale / Laurea magistrale / Dottorato di ricerca*	**Highest level of education**<br>*Response options: Primary school / Lower secondary school / Upper secondary school diploma / Bachelor's degree / Master's degree / Doctoral degree*
**Q3**	**A**	**Età**<br>*Response options: 18–24 / 25–34 / 35–44 / 45–54 / 55–64 / 65–74 / ≥75*	**Age group**<br>*Response options: 18–24 / 25–34 / 35–44 / 45–54 / 55–64 / 65–74 / ≥75*
**Q4**	**A**	**Provincia di residenza**<br>*Response options: Città metropolitana di Cagliari / Sud Sardegna / Oristano / Nuoro / Sassari / Sono residente fuori Sardegna ma di origini sarde / Nessuna di queste*	**Province of residence**<br>*Response options: Metropolitan City of Cagliari / South Sardinia / Oristano / Nuoro / Sassari / Resident outside Sardinia but of Sardinian origin / None of the above*
**Q5**	**A**	**Risiedi in Sardegna da più di 10 anni?**<br>*Response options: Sì / No*	**Have you been living in Sardinia for more than 10 years?**<br>*Response options: Yes / No*
**Q6**	**A**	**Quali sono le tue origini?**<br>*Response options: Mediterranea (es. Sud Italia, Sardegn ecc.) / Altra*	**What are your ethnic/geographic origins?**<br>*Response options: Mediterranean (e.g. Southern Italy, Sardini etc.) / Other*
**Q7**	**A**	**Sei fabico/carente di G6PD?**<br>*Response options: Sì / No / Non so*	**Are you G6PD-deficient (do you have favism)?**<br>*Response options: Yes / No / I don't know*

Item	Cat.	Italian (original)	English (translation)
Q8	A	**Solo per le femmine carenti (non obbligatoria): presenti carenza intermedia o totale?**<br>*Response options: Carenza intermedia / Carenza totale / Non so*	**For G6PD-deficient females only (optional): do you have intermediate or total enzyme deficiency?**<br>*Response options: Intermediate deficiency / Total deficiency / I don't know*
Q9	A	**Qualcuno nella tua famiglia ha il favismo/carenza di G6PD?**<br>*Response options: Sì / No / Non so*	**Does anyone in your family have favism/G6PD deficiency?**<br>*Response options: Yes / No / I don't know*
Q10	A/B/C	**Secondo te, quali sono i comportamenti da evitare per un fabico? (risposta multipla)**<br>*Response options: Assunzione di fave / Assunzione di piselli / Assunzione di altri legumi / Utilizzo di farmaci specifici (es. alcuni antimalarici) / Utilizzo di henné / Farsi tatuaggi / Respirare polline delle fave*	**In your opinion, which of the following should a person with G6PD deficiency avoid? (multiple-choice)**<br>*Response options: Eating fava beans [A] / Eating peas [C] / Eating other legumes [C] / Taking specific medications (e.g. some antimalarials) [A] / Using henna [B] / Getting tattoos [C] / Inhaling fava bean pollen [B]*
Q11	A	**Hai mai avuto una crisi emolitica da ingestione di fave?**<br>*Response options: Sì / No / Non so*	**Have you ever had a haemolytic crisis after eating fava beans?**<br>*Response options: Yes / No / I don't know*
Q12	B	**Sei mai stato male dopo aver sentito l'odore di fave cotte o crude o respirato il polline durante la fioritura delle fave?**<br>*Response options: Sì / No / Non so*	**Have you ever felt unwell after smelling raw or cooked fava beans, or after inhaling fava bean pollen during the flowering season?**<br>*Response options: Yes / No / I don't know*
Q13	B	**Che sintomi hai avuto dopo aver sentito l'odore delle fave o respirato il polline? (risposta multipla)**<br>*Response options: Sensazione di fastidio rispetto all'odore percepito / Sintomi allergici (prurito, rossore, starnuti ecc.) / Pallore / Nausea / Cefalea / Debolezza / Anemia / Urine scure (color marsala) / Dolori addominali / Nessuno di questi sintomi*	**What symptoms did you experience after fava bean odour/pollen exposure? (multiple-choice)**<br>*Response options: Discomfort related to the perceived odour / Allergic symptoms (itching, redness, sneezing, etc.) / Pallor / Nausea / Headache / Weakness / Anaemia / Dark urine (Marsala-coloured) / Abdominal pain / None of these symptoms*
Q14	A	**Hai mai assunto uno dei seguenti farmaci prima di avere sintomi sospetti come quelli elencati in precedenza?**<br>*Response options: Aspirina / Antibiotici sulfamidici / Antimalarici / Altri farmaci / Non ho mai avuto sintomi sospetti dopo l'utilizzo di farmaci*	**Have you ever taken any of the following medications before experiencing symptoms similar to those listed above?**<br>*Response options: Aspirin / Sulfonamide antibiotics / Antimalarials / Other drugs / I have never had suspicious symptoms after taking any medication*

Item	Cat.	Italian (original)	English (translation)
Q15	A	**Dove hai ricevuto le informazioni che possiedi sul favismo?** *Response options: Scuola / Università / Siti internet e social network / Medico di famiglia o medici in strutture ospedaliere / In famiglia o tramite amici*	**Where did you receive information about favism? (multiple-choice)** *Response options: School / University / Websites and social media / General practitioner or hospital doctors / Family or friends*
Q16	A	**Ritieni venga fatta abbastanza divulgazione in merito al favismo, a come si trasmette geneticamente e a quali comportamenti tenere per evitare pericoli per la salute?** *Response options: Sì / Più Sì che No / Più No che Sì / No*	**Do you think enough public information is provided about favism, its genetic transmission, and the behaviours to follow to avoid health risks?** *Response options: Yes / More yes than no / More no than yes / No*

** Q8 was displayed only to respondents who answered 'Yes' (Sì) to Q7. It was explicitly labelled as non-mandatory.*

Abbreviations: G6PD = glucose-6-phosphate dehydrogenase; Cat. = evidence category (A/B/C — see legend above).

Supplementary Material S2

STROBE Checklist for Cross-Sectional Studies

Reference: Vonghia E. et al. (2025). Checklist based on: Vandenbroucke JP et al. Strengthening the Reporting of Observational Studies in Epidemiology (STROBE): Explanation and Elaboration. PLoS Med. 2007;4(10):e297.

Item No.	Recommendation	Reported (Y/N/NA)	Location in manuscript
**TITLE AND ABSTRACT**			
1	(a) Indicate the study's design with a commonly used term in the title or the abstract. (b) Provide in the abstract an informative and balanced summary of what was done and what was found.	Yes	Title ("cross-sectional survey"); Abstract, lines 1–12
**INTRODUCTION**			
2	Background/rationale: Explain the scientific background and rationale for the investigation being reported.	Yes	Section 1 (Introduction), paragraphs 1–4
3	Objectives: State specific objectives, including any pre-specified hypotheses.	Yes	Section 1 (Introduction), final paragraph
**METHODS**			
4	Study design: Present key elements of study design early in the paper.	Yes	Section 2.1 (Study Design and Participants)

Item No.	Recommendation	Reported (Y/N/NA)	Location in manuscript
5	Setting: Describe the setting, locations, and relevant dates, including periods of recruitment, exposure, follow-up, and data collection.	Yes	Section 2.1; recruitment conducted in Sardinia, Italy; survey period stated
6	Participants: (a) Give the eligibility criteria, and the sources and methods of selection of participants.	Yes	Section 2.1 (inclusion criteria: Sardinian residents; online convenience sample)
7	Variables: Clearly define all outcomes, exposures, predictors, potential confounders, and effect modifiers. Give diagnostic criteria, if applicable.	Yes	Section 2.2 (Questionnaire); outcome = perceived adequacy of information; predictors = age, gender, G6PD status
8	Data sources/measurement: For each variable of interest, give sources of data and details of methods of assessment (measurement). Describe comparability of assessment methods if there is more than one group.	Yes	Section 2.2; self-administered structured questionnaire described in detail
9	Bias: Describe any efforts to address potential sources of bias.	Yes	Section 4.5 (Limitations); affected-network enrichment, recall bias, and selection bias discussed
10	Study size: Explain how the study size was arrived at.	Partial	Section 2.1; convenience sample of 536 respondents; no formal power calculation performed (acknowledged in limitations)
11	Quantitative variables: Explain how quantitative variables were handled in the analyses. If applicable, describe which groupings were chosen and why.	Yes	Section 2.3; age treated as 7-level ordinal variable; groupings follow standard epidemiological bands

Item No.	Recommendation	Reported (Y/N/NA)	Location in manuscript
12	Statistical methods: (a) Describe all statistical methods, including those used to control for confounding. (b) Describe any methods used to examine subgroups and interactions. (c) Explain how missing data were addressed. (d) If applicable, describe analytical methods taking account of sampling strategy. (e) Describe any sensitivity analyses.	Yes	Section 2.3 (Statistical Analysis); chi-square, Cochran-Armitage trend test, logistic regression with OR and 95% CI; missing data reported (n=2 non-responders)
**RESULTS**			
13	Participants: (a) Report numbers of individuals at each stage of study — e.g. numbers potentially eligible, examined, confirmed eligible, included in the study, completing follow-up, and analysed. (b) Give reasons for non-participation at each stage. (c) Consider use of a flow diagram.	Yes	Section 3.1; n=536 total respondents; n=534 for adequacy item (2 missing responses noted); Figure 1
14	Descriptive data: (a) Give characteristics of study participants (e.g. demographic, clinical, social) and information on exposures and potential confounders. (b) Indicate number of participants with missing data for each variable of interest.	Yes	Section 3.1; Table 1–3; demographic characteristics by G6PD stratum; missing data reported per item
15	Outcome data: Report numbers of outcome events or summary measures.	Yes	Section 3.2; Table 4–5; perceived adequacy rates by age, gender, and G6PD status fully reported
16	Main results: (a) Give unadjusted estimates and, if applicable, confounder-adjusted estimates and their precision (e.g. 95% confidence interval). Make clear which confounders were adjusted for and why they were included. (b) Report category boundaries when continuous variables were categorized. (c) If relevant, consider translating estimates of relative risk into absolute risk for a meaningful time period.	Yes	Table 5C; crude ORs with 95% CI reported; Cochran-Armitage Z and logistic regression OR per age-band reported in text
17	Other analyses: Report other analyses done — e.g. analyses of subgroups and interactions, and sensitivity analyses.	Yes	Section 3.2; stratified analyses by G6PD status, age group, and gender reported; small-subgroup limitations acknowledged
**DISCUSSION**			

Item No.	Recommendation	Reported (Y/N/NA)	Location in manuscript
18	Key results: Summarise key results with reference to study objectives.	Yes	Section 4.2; key findings on perceived inadequacy, age gradient, and gender difference summarised
19	Limitations: Discuss limitations of the study, taking into account sources of potential bias or imprecision. Discuss both direction and magnitude of any potential bias.	Yes	Section 4.5 (Limitations); convenience sampling, self-report bias, lack of biochemical confirmation, small subgroups, unvalidated questionnaire all discussed
20	Interpretation: Give a cautious overall interpretation of results considering objectives, limitations, multiplicity of analyses, results from similar studies, and other relevant evidence.	Yes	Sections 4.2–4.4; results interpreted with explicit acknowledgment of sample limitations; comparisons with prior literature included
21	Generalisability: Discuss the generalisability (external validity) of the study results.	Yes	Sections 4.1, 4.4, 4.5; convenience sample explicitly described as non-representative; applicability to other G6PD-endemic regions discussed
**OTHER INFORMATION**			
22	Funding: Give the source of funding and the role of the funders for the present study and, if applicable, for the original study on which the present article is based.	Yes	Funding statement: "This research received no external funding"

Note: *"Partial" indicates that the element is addressed but incompletely (e.g. no formal power calculation). All items judged as not applicable (NA) are explained in the corresponding cell. Section and page numbers refer to the final proof version of the manuscript.*
